# Randomized control trial comparing an Alvarado Score-based management algorithm and current best practice in the evaluation of suspected appendicitis

**DOI:** 10.1186/s13017-020-00309-0

**Published:** 2020-05-01

**Authors:** Winson Jianhong Tan, Sanchalika Acharyya, Min Hoe Chew, Fung Joon Foo, Weng Hoong Chan, Wai Keong Wong, London Lucien Ooi, Jeremy Chung Fai Ng, Hock Soo Ong

**Affiliations:** 1 Department of General Surgery, Sengkang General Hospital, Singapore, Singapore; 2 Department of Colorectal Surgery, Singapore General Hospital, Singapore, Singapore; 3 Clinical Research & Innovation Office, Tan Tock Seng Hospital Singapore, Singapore, Singapore; 4 Department of General Surgery, Singapore General Hospital, Singapore, Singapore

**Keywords:** Appendicitis, Algorithm, Alvarado score, CT utilization

## Abstract

**Background:**

An objective algorithm for the management of suspected appendicitis guided by the Alvarado Score had previously been proposed. This algorithm was expected to reduce computed tomography (CT) utilization without compromising the negative appendectomy rate. This study attempts to validate the proposed algorithm in a randomized control trial.

**Methods:**

A randomized control trial comparing the management of suspected acute appendicitis using the proposed algorithm compared to current best practice, with the rate of CT utilization as the primary outcome of interest. Secondary outcomes included the percentage of missed diagnosis, negative appendectomies, length of stay in days, and overall cost of stay in dollars.

**Results:**

One hundred sixty patients were randomized. Characteristics such as age, ethnic group, American Society of Anesthesiologist score, white cell count, and symptom duration were similar between the two groups. The overall CT utilization rate of the intervention arm and the usual care arm were similar (93.7% vs 92.5%, *p* = 0.999). There were no differences in terms of negative appendectomy rate, length of stay, and cost of stay between the intervention arm as compared to the usual care arm (*p* = 0.926, *p* = 0.705, and *p* = 0.886, respectively). Among patients evaluated with CT, 75% (112 out of 149) revealed diagnoses for the presenting symptoms.

**Conclusion:**

The proposed AS-based management algorithm did not reduce the CT utilization rate. Outcomes such as missed diagnoses, negative appendectomy rates, length of stay, and cost of stay were also largely similar. CT utilization was prevalent as 93% of the study cohort was evaluated by CT scan.

**Trial registration:**

The study has been registered at ClinicalTrials.gov (NCT03324165, Registered October 27 2017).

## Background

Acute appendicitis is one of the most common causes of acute abdominal pain requiring surgical intervention, with a lifetime risk of 8.6% for males and 6.7% for females [1, 2]. Historically, negative appendectomy rates of more than 20% were considered the norm. However, this may no longer be acceptable in the current era as even though complication rates in the setting of negative appendectomy are low, conditions such as incisional hernias, intestinal obstruction secondary to adhesions, and stump leakages can result in significant morbidity [3, 4].

Computed tomography (CT) scan has emerged as the dominant imaging modality for the evaluation of suspected appendicitis in adults [5]. It has decreased negative appendectomy rates and has sensitivity and specificity of 95% and 94%, respectively, based on data from a recent meta-analysis [6]. However, the radiation exposure with CT poses a concern, particularly in appendicitis, which occurs predominantly in young patients most susceptible to the adverse effects of radiation [7, 8]. Available literature has estimated that at least 25% of CT scans are not clinically warranted and may pose more harm than benefits [9]. Rules for clinical decision guiding CT utilization is thus essential to minimize unnecessary CT scans.

**Fig. 1:**
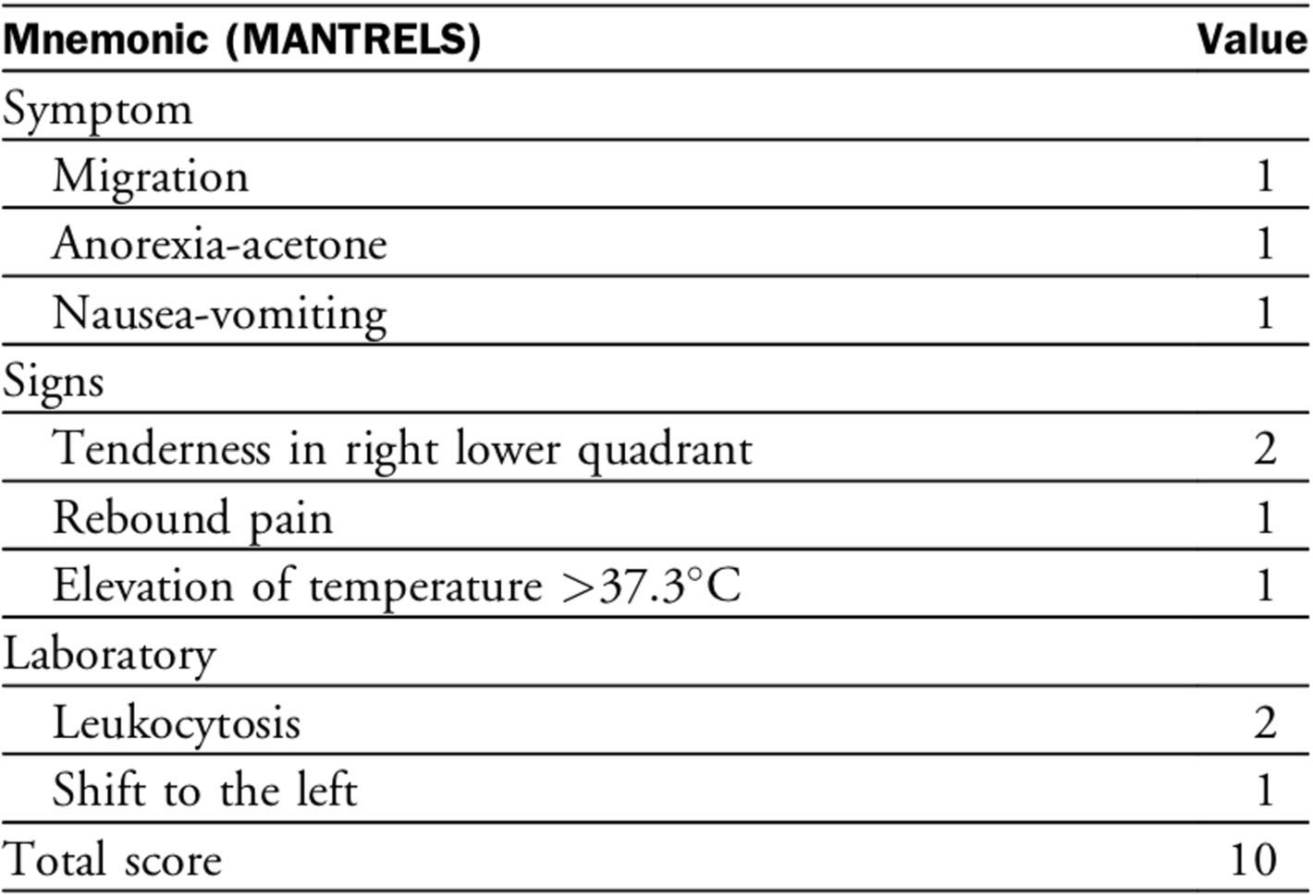
Alvarado Score for acute appendicitis

**Fig. 2:**
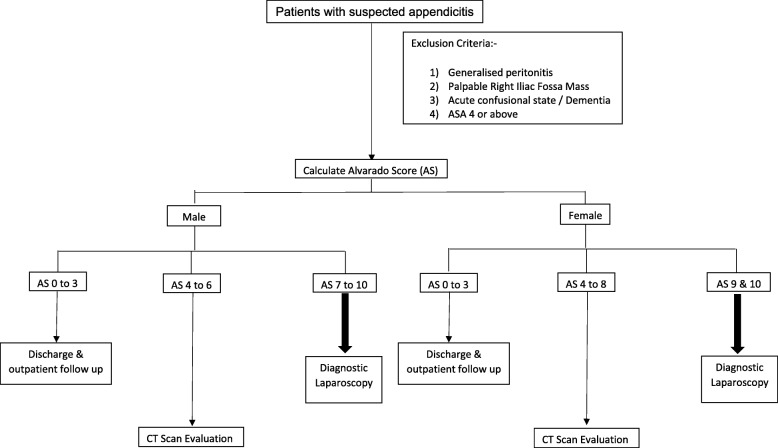
Proposed algorithm for the management of suspected appendicitis guided by the Alvarado Score

Currently, the management of suspected appendicitis is surgeon dependent. Accuracy of diagnosis is dependent on individual’s clinical acumen, preference for CT scan, and threshold for offering surgery. The Alvarado Score (AS) is a 10-point clinical scoring system for acute appendicitis that has been extensively validated (Fig. 1) [10, 11]. In a prior publication, we have prospectively validated the AS on 500 consecutive cases of suspected appendicitis [12]. Comparing the diagnostic performance measures of CT scan with the AS, we have identified ranges of AS where patients are unlikely to benefit from CT evaluation. Based on these findings, we have formulated an objective algorithm for the management of suspected appendicitis guided by the AS (Fig. 2) [12]. We believe this algorithm can guide CT utilization and reduce the number of CT scans ordered with an acceptable negative appendectomy rate.

Hence, we attempt to validate this proposed algorithm in a randomized control trial.

## Methods

A randomized control trial comparing the management of suspected acute appendicitis using the proposed algorithm (Fig. 2) compared to the current best practice with the rate of CT utilization as the primary outcome of interest. The study has been registered at ClinicalTrials.gov (NCT 03324165).

### Participants

Patients were recruited from the Acute Care Surgery Service of Singapore General Hospital (1500-bed general hospital) and Sengkang General Hospital (1000-bed general hospital). The target population consisted of patients between the ages of 16 and 80 who were admitted to the General Surgery department of either hospitals with a diagnosis of suspected appendicitis.

Patients who were pregnant and had generalized peritonitis or a palpable mass on presentation were excluded from the study. Other exclusion criteria were age less than 16 years or more than 80 years old, evidence of delirium or dementia, high risk for surgery (American Society of Anesthesiologists score of 4 and above), and immunocompromised state.

Informed consent was obtained from patients prior to formal recruitment into the study.

### Randomization

Patients were randomized into one of the two management arms at the point of initial assessment by the surgical team.

i) Intervention arm—Computation of the AS with management as per proposed algorithm (Fig. 2)
ii) Usual care arm—Current best practice (based on the discretion of the attending surgeon)

Patients were randomized into either the intervention or usual care arm in equal numbers (*n* = 80). The randomization schedule was generated using standard statistical software by a statistician who was not involved in data analysis. Envelopes containing the treatment instructions were marked according to that schedule. Randomization was performed in blocks of six subjects, three for the intervention and three for the control arm, to ensure balanced groups.

### Outcome parameters

The primary outcome of interest was the CT utilization rate between the intervention and usual care arm. Secondary outcomes included the percentage of missed diagnosis, negative appendectomies, length of stay in days, and overall cost of stay in dollars.

The definitions of the above outcome measures were as follows:

i) CT utilization—The proportion of patients with CT scans performed within each management arm
ii) Missed diagnosis—Patients who were not diagnosed with acute appendicitis during the initial admission but were subsequently readmitted within 2 weeks of discharge due to progression of symptoms, with eventual surgery showing acute appendicitis on histology
iii) Negative appendectomies—Patients who were operated with a pre-operative diagnosis of acute appendicitis with subsequent histology showing no features of acute appendicitis
iv) Length of stay—Duration of total hospitalization (measured in days) from point of admission to discharge during the study follow-up period
v) Cost of stay—Total cost incurred by the patient in Singapore dollars during admission. This includes ward charges, medications, and costs of diagnostic procedures

### Data collection and follow-up

Pre, per-, and post-treatment data were collected prospectively in a standardized data collection sheet. Study data was collected and managed using the REDCap electronic data capture tools hosted at Singapore General Hospital. REDCap (Research Electronic Data Capture) is a secure, Web-based application designed to support data capture for research studies [13].

Patients who were randomized to the control arm had their AS retrospectively calculated by the study coordinator to allow comparison between the various AS categories.

In patients who underwent CT evaluation, the eventual results were categorized into 1 of the following 4 categories by the attending surgeon.

i) Acute appendicitis diagnosed on CT scan
ii) No acute appendicitis but alternative diagnosis for symptoms established. These alternative diagnoses include bowel pathology (colitis/enteritits, diverticulitis, colonic malignancy), urologic pathology (urinary tract infections and ureteric calculi), and gynaecologic conditions such as pelvic inflammatory disease, ruptured ovarian cysts, or ovarian torsion
iii) No acute appendicitis without any alternative diagnosis for symptoms established
iv) Equivocal for acute appendicitis

Patients were followed-up in person 2 weeks after discharge. Subsequent follow-up was determined based on clinical indication. Patients lost to follow-up were contacted via the phone to determine if an initial diagnosis of appendicitis had been missed. A search via the National Electronic Health Record (NEHR) database was also performed to identify patients who defaulted but re-presented at another hospital for treatment. The NEHR database captures the admission information of every person who has visited the public health care system in Singapore.

### Sample size calculation

Based on our previous publication, 80% of all patients with suspected appendicitis were subjected to CT evaluation [12]. If the algorithm had been implemented on this group of patients, the percentage of CT scans performed could have been reduced to 60%. To determine the specified difference in proportion of CT scan utilization using a one-sided chi-square test assuming 80% power and 5% type I error rate, 160 patients (80 subjects with suspected appendicitis in each study arm) were required after accounting for 10% lost to follow-up.

### Analysis plan

Study data was analyzed using appropriate summary statistics and statistical tests to address the study objectives. The distribution of baseline demographics and clinical characteristics by study arm was summarized.

The proportion of CT utilization, the primary outcome of interest, was compared between the two study arms using a one-sided chi-square test.

The CT utilization rate, stratified by the AS category, was compared between the two study arms using Fisher’s exact test. As there were relatively few patients with AS of 1, 2, and 3, these were collated into one category (AS 1 to 3) during the above analysis.

Negative appendectomy rate and proportion of missed diagnosis along with a 95% confidence interval were estimated for each study arm using simple asymptotic methods. Median length and cost of stay between the 2 study arms were compared using the Mann-Whitney *U* test. All statistical analysis was done using R 3.1.1 (R Core Tam 2014, Vienna, Austria), and statistical significance was taken as *P* value < 0.05. This study was carried out under the approval of the Centralized Institutional Review Board of the Singapore Health Services.

## Results

**Fig. 3:**
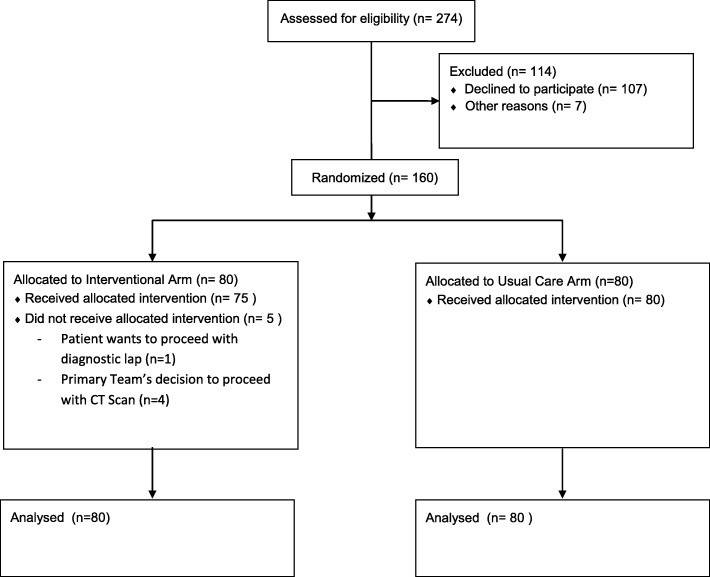
CONSORT flow diagram of the study cohort

Figure 3 illustrates the CONSORT flow diagram for the trial. From October 2017 to May 2018, a total of 274 patients were screened of which 160 underwent subsequent randomization. One hundred fourteen patients were excluded from the study; 7 failed to meet the inclusion criteria (2 patients less than 16 years of age, 3 patients more than 80 years of age, and 2 patients had dementia) while the remaining 107 patients refused to participate in the study. The baseline characteristics of those who declined to participate in the study were similar to those who underwent randomization in terms of gender, age, and white cell count.

The baseline demographic characteristics of the 2 groups were illustrated in Table 1. There appears to be a higher proportion of female patients in the intervention arm of the study (70% vs 56.3%) even though the difference was not statistically significant (*p* = 0.101). Characteristics such as age, ethnic group, American Society of Anesthesiologist score, white cell count, and symptom duration were otherwise similar between the two groups. In terms of AS distribution, there was a lower proportion of patients with Alvarado Score of < 3 in the intervention arm as compared to the usual care arm (3.8% vs 15.1%, *P* = 0.041).

**Table 1:** Baseline demographics of the study cohort

	Intervention arm (*n* = 80)	Usual care arm (*n* = 80)	*P* value
Gender (%)			0.101
Male	24 (30.0)	35 (43.8)	
Female	56 (70.0)	45 (56.3)	
Ethnic group			0.099
Chinese	35 (43.8)	43 (53.8)	
Malay	17 (21.3)	23 (28.8)	
Indian	15 (18.8)	9 (11.3)	
Others	13 (16.3)	5 (6.3)	
Mean age ± SD	38.2 ± 13.7	38.7 ± 12.8	0.830
ASA score			0.888
ASA 1—Normal healthy patient	32 (40.0)	35 (43.8)	
ASA 2—Mild systemic disease	45 (56.3)	42 (52.5)	
ASA 3—Severe systemic disease	3 (3.8)	3 (3.8)	
White cell count, × 10^9^/L, mean (SD)	11.8 ± 3.8	12.4 ± 4.9	0.350
Duration of symptoms (days)	2.7 ± 2.3	2.6 ± 1.7	0.788
Alvarado Score Distribution			0.041
1	0 (0.0)	1 (1.3)	
2	1 (1.3)	2 (2.5)	
3	2 (2.5)	9 (11.3)	
4	15 (18.8)	14 (17.5)	
5	24 (30.0)	11 (13.8)	
6	15 (18.8)	16 (20.0)	
7	15 (18.8)	11 (13.8)	
8	8 (10.0)	12 (15.0)	
9	0 (0.0)	4 (5.0)	
10	0 (0.0)	0 (0.0)	
Surgery performed	32 (40.0)	30 (37.5)	0.871
Types of surgery performed			0.468
Open appendectomy	2 (2.5)	0 (0.0)	
Laparoscopic appendectomy	25 (31.3)	22 (27.5)	
Diagnostic laparoscopy	4 (5.0)	5 (6.3)	
Others	1 (1.3)	3 (3.8)	

The characteristics of CT scan utilization for the 2 groups are illustrated in Table 2. The overall CT utilization rate of the intervention arm and the usual care arm were similar (93.7% vs 92.5%, *P* = 0.999). When CT scan utilization was stratified by the various AS categories, utilization rate among patients with AS 1–3 was significantly lower in the intervention arm than the usual care arm (33.3% versus 83.3%, *P* = 0.012).

**Table 2:** Computed tomography scan utilization among various Alvarado Score categories

		Overall (*n* = 160)	Intervention arm (*n* = 80)	Usual care arm (*n* = 80)	*P* value
Overall CT Utilization		149 (93.1)	75 (93.7)	74 (92.5)	0.999
CT scans performed stratified by Alvarado Score Category					
Alvarado Score	Number of patients in intervention arm	Number of CT scans done (%)	Number of patients in usual care arm	Number of CT scans done (%)	
1 to 3	3	1 (33.3)	12	10 (83.3)	0.012
4	15	15 (100)	14	13 (92.9)	
5	24	23 (95.8)	11	10 (90.9)	
6	15	14 (93.3)	16	16 (100)	
7	15	15 (100)	11	10 (90.9)	
8	8	7 (87.5)	12	12 (100)	
9	0		4	3 (75)	

Secondary outcome measures were illustrated in Table 3. There was no missed diagnosis in either of the study arms. There were also no differences in terms of negative appendectomy rates and length of stay between the intervention arm as compared to the usual care arm (*P* = 0.926 and *P* = 0.705, respectively). The cost of stay between both arms of the study was similar with a median cost of $3973 in the intervention arm and $3703 in the usual care arm (*P* = 0.886).

**Table 3:** Comparison of secondary outcome measures

	Intervention arm (*n* = 80)	Usual care arm (*n* = 80)	*P* value
Missed diagnosis	0 (0.0)	0 (0.0)	
Histological-proven appendicitis among the subjects who had surgery*	28 (87.5)	27 (90.0)	0.866
Negative appendectomy among the subjects who had surgery*	4 (12.5)	3 (10)	0.926
Length of stay in days			
Median (range)	2 (1–14)	2 (0–13)	0.705
Gross cost of stay (S$)			
Median (range)	3973 (2092–26009)	3703 (1724–27321)	0.886

*Percentages calculated using number of patients who underwent surgery in each arm (32 in intervention arm, 30 in the control arm) as denominator

The distribution of CT scan findings for the entire study cohort is illustrated in Table 4. Acute appendicitis was diagnosed in 31.5% of the CT scans performed while 43.6% of the scans excluded acute appendicitis and provided an alternative diagnosis for the patient’s presenting symptoms. Equivocal CT scan findings accounted for 2% of all the CT scans performed.

**Table 4:** Distribution of CT Scan findings

Results	Overall (*n* = 149)
Acute appendicitis	47 (31.5)
No acute appendicitis but alternative diagnosis for symptoms established	65 (43.6)
Acute appendicitis excluded with no alternative diagnosis established	34 (22.8)
Equivocal	3 (2.0)

## Discussion

The proposed management algorithm failed to demonstrate any difference in terms of its primary outcome of a reduction in CT utilization (93.7% vs 92.5%, *P* = 0.999). Neither were there differences demonstrated in terms of the secondary outcomes of missed diagnoses, negative appendectomy rates, length of stay, and cost of stay. It was apparent that in our practice, CT scans are considered the mainstay of evaluation for suspected appendicitis, as more than 90% of patients were subjected to CT evaluation both in the algorithm arm and the usual care arm.

The reason why CT utilization was not reduced with the management algorithm becomes apparent upon analysis of the AS score distribution of patients randomized to the algorithm arm of the study. The algorithm hinges on management decisions for patients with AS 3 and below (discharge to home) and AS of 7 and above in males and 9 and above in females (diagnostic laparoscopy) to reduce CT utilization. In the algorithm arm of the study, only 6 out of 80 (7.5%) fell within this category where management decisions were supposed to be made without further CT evaluation. In comparison, the corresponding proportion was 27 out of 80 (33.8%) patients in the usual care arm. Furthermore, among the 6 patients within the algorithm arm of the study who were supposed to be managed without CT evaluation, 4 out of the 6 had CT performed due to the preference of the attending surgeon. Surgeon preference may have influenced the effectiveness of the algorithm in reducing CT utilization as components of the AS such as presenting symptoms of migratory pain, anorexia, nausea/vomiting, and physical findings of right iliac fossa tenderness or rebound pain all comprise a subjective element. Hence, an attending surgeon’s bias for CT evaluation may be satisfied by scoring patients within AS score categories in which CT evaluation would be performed.

In any management decisions, patient’s preference ought to be considered and some may prefer CT evaluation to minimize any risks of unnecessary surgery [14]. Estimates have indicated that complications can occur in 12% of normal appendectomies [4]. It has been estimated that every 100 CT scans performed for suspected appendicitis prevented 21 unnecessary operations [15]. These statistics have prompted some to recommend routine CT evaluation for all patients who present with suspected appendicitis [16, 17]. Analysis of our recruitment data suggests that such an approach may be preferred by certain patients. During patient recruitment, 107 out of 274 patients refused to participate in the study. Further analysis on the reasons for refusal revealed that more than 80% rejected participation as they were keen for CT evaluation to mitigate the risk of unnecessary surgery.

Despite concerns regarding radiation exposure, CT evaluation is likely to remain as the main diagnostic modality for the evaluation of suspected appendicitis. In our study, CT evaluation established the diagnosis of acute appendicitis, or alternative diagnoses mimicking appendicitis, in 75% of cases. Ultrasound has been proposed as an alternative for initial evaluation and is included in the evaluation algorithm proposed by the World Society of Emergency Surgery [18]. However, it has not taken off in our practice as the findings are operator dependent. Results from a recent meta-analysis have also concluded that the sensitivity and specificity of ultrasound were not superior to that of clinical examination [19]. While magnetic resonance imaging of the abdomen is a reasonable alternative, its rate of a non-diagnostic exam is higher than that of CT [20]. Magnetic resonance imaging is also more costly and less tolerated by patients.

Perhaps, efforts should be dedicated towards making CT evaluation safer rather than trying to replace it. Limited range CT (from top of L2 to the symphysis pubis) has shown promise among the pediatric population with close to 50% reduction in exposed radiation [21]. However, its use may not be feasible in the adult population as several mimicking pathologies such as diverticulitis and upper track urinary infections may not be confined to the pelvis and may hence be missed with limited range CT [22]. Low-dose CT (2-4 mSv) is a more promising alternative with recent studies indicating performance characteristics comparable to those of standard dose CT (8-10 mSv) with less than half of exposed radiation [23, 24].. This may make the routine use of CT evaluation for all cases of suspected appendicitis more palatable for some clinicians.

The main limitation of our study was the absence of blinding which meant that clinicians’ inherent biases towards CT evaluation could not be eliminated. However, blinding in our study design was impossible as the attending clinician had to be aware of the allocated study arm to decide if the AS needed to be calculated to guide subsequent management. In addition, the study was performed on patients recruited and managed in 2 institutions which limits the generalizability of the study findings. Nonetheless, this was a novel study evaluating an AS based management algorithm to guide CT utilization. While the proposed algorithm failed to reduce CT utilization rate, CT evaluation appears to be preferred by most surgeons as CT utilization exceeded 90% in both study arms.

## Conclusion

The proposed AS-based management algorithm did not reduce the CT utilization rate. Outcomes such as missed diagnoses, negative appendectomy rates, length of stay, and cost of stay were also largely similar. CT evaluation appears to be preferred by most surgeons as 93% of the study cohort with suspected appendicitis was evaluated by CT scan.

## Data Availability

The dataset analyzed during the current study is available from the corresponding author on a reasonable request.
